# INNOVATIVE POLICIES AND TECHNOLOGIES TO INCREASE ACCESS TO HEARING AIDS FOR ADULTS

**DOI:** 10.1093/geroni/igz038.3053

**Published:** 2019-11-08

**Authors:** Michael Yong, Amber Willink, Catherine McMahon, Bradley McPherson, Carrie L Nieman, Nicholas Reed, Frank Lin

**Affiliations:** 1 Johns Hopkins University, Baltimore, Maryland, United States; 2 Macquarie University, Sydney, Australia; 3 The University of Hong Kong, Hong Kong, China; 4 Johns Hopkins University School of Medicine, Department of Otolaryngology-HNS, Baltimore, Maryland, United States

## Abstract

As the proportion of older adults in the world’s total population continues to grow, the deleterious downstream health economic outcomes of age-related hearing loss are steadily becoming more prevalent. While recent research has shown that age-related hearing loss is the single greatest modifiable risk factor for dementia, the rate of hearing aid use remains low in many countries across the globe. Reasons for poor uptake are multifactorial and likely involve a combination of factors, ranging from increasing costs of hearing aid technology to lack of widespread insurance coverage. This paper aims to first identify the current state of hearing aid access across the world using eight representative countries as examples. We then provide recommendations on how to facilitate greater access to hearing aids for consumers by addressing areas in regulation, technology, reimbursement, and workforce.

